# The Decisive Case-Control Study Elaborates the Null Association between *ESR1* XbaI and Osteoarthritis in Asians: A Case–Control Study and Meta-Analysis

**DOI:** 10.3390/genes12030404

**Published:** 2021-03-12

**Authors:** Yu-Hao Huang, Wen-Hui Fang, Dung-Jang Tsai, Yu-Hsuan Chen, Yu-Chiao Wang, Wen Su, Chung-Cheng Kao, Kevin Yi, Chih-Chien Wang, Sui-Lung Su

**Affiliations:** 1 Graduate Institute of Medical Sciences, National Defense Medical Center, Taipei 11490, Taiwan; zeynepeason@gmail.com; 2 Department of Family and Community Medicine, Tri-Service General Hospital, National Defense Medical Center, Taipei 11490, Taiwan; rumaf.fang@gmail.com; 3 Graduate Institute of Life Sciences, National Defense Medical Center, Taipei 11490, Taiwan; oo800217@gmail.com; 4 School of Public Health, National Defense Medical Center, Taipei 11490, Taiwan; tamago1117@gmail.com (Y.-H.C.); onlyndmcbest@gmail.com (Y.-C.W.); 5 Department of Education & Medical Research, Taoyuan Armed Forces General Hospital, Taoyuan 325, Taiwan; 6 Graduate Institute of Aerospace and Undersea Medicine, National Defense Medical Center, Taipei 11490, Taiwan; suwen9319@gmail.com; 7 Superintendent’s Office, Tri-Service General Hospital Songshan Branch, National Defense Medical Center, Taipei 10581, Taiwan; kao8267kq@gmail.com; 8 Department of Biochemistry, Purdue University, West Lafayette, IN 47907-2063, USA; yi77@purdue.edu; 9 Department of Orthopedics, Tri-Service General Hospital and National Defense Medical Center, Taipei 11490, Taiwan

**Keywords:** estrogen receptor 1 (*ESR1*), osteoarthritis (OA), rs9340799, *ESR1* XbaI, polymorphism, meta-analysis, trial sequential analysis

## Abstract

(1) Background: The prevalence of knee osteoarthritis (OA) in women is significantly higher than in men. The estrogen receptor α (ERα) has been considered to play a key role due to a large gender difference in its expression. ERα is encoded by the gene estrogen receptor 1 (*ESR1*), which is widely studied to explore the gender difference in knee OA. Several polymorphisms in *ESR1* [PvuII (rs2234693) and BtgI (rs2228480)] were confirmed as the risk factors of OA. However, the evidence of the last widely investigated polymorphism, *ESR1* Xbal (rs9340799), is still insufficient for concluding its effect on knee OA. (2) Objective: This study proposed a case–control study to investigate the association between *ESR1* Xbal and knee OA. Moreover, a meta-analysis and trial sequential analysis (TSA) were conducted to enlarge the sample size for obtaining a conclusive evidence. (3) Methods: In total, 497 knee OA cases and 473 healthy controls were recruited between March 2015 and July 2018. The Kellgren–Lawrence grading system was used to identify the knee OA cases. To improve the evidence level of our study, we conducted a meta-analysis including the related studies published up until December 2018 from PubMed, Embase, and previous meta-analysis. The results are expressed as odds ratios (ORs) with corresponding 95% confidence intervals (CI) for evaluating the effect of this polymorphism on knee OA risk. TSA was used to estimate the sample sizes required in this issue. (4) Results: We found non-significant association between the G allele and knee OA [Crude-OR: 0.97 (95% CI: 0.78–1.20) and adjusted-OR: 0.90 (95% CI: 0.71–1.15) in allele model] in the present case–control study, and the analysis of other genetic models showed a similar trend. After including six published studies and our case–control studies, the current evidence with 3174 Asians showed the conclusively null association between *ESR1* XbaI and knee OA [OR: 0.78 (95% CI: 0.59–1.04)] with a high heterogeneity (*I^2^*: 78%). The result of Caucasians also concluded the null association [OR: 1.05 (95% CI: 0.56–1.95), *I^2^*: 87%]. (5) Conclusions: The association between *ESR1* XbaI and knee OA was not similar with other polymorphisms in *ESR1*, which is not a causal relationship. This study integrated all current evidence to elaborate this conclusion for suggesting no necessity of future studies.

## 1. Introduction

Knee osteoarthritis (OA) is a leading cause of disability in most developed countries [1,2,3,4]. Genetic factors play a key role in knee OA pathogenesis. The heritability of knee OA was estimated as 65% [5,6]. Moreover, previous research suggested that OA is primarily influenced by genetic risk factors due to common population polymorphisms in multiple genes [7,8,9]. It is desirable to identify more candidate genes and evaluate their effects.

The epidemiological findings show that OA is more common in women than men [10], especially after menopause [11,12]. Women are more likely than men to show a deactivation or alteration of miRNAs that are important for estrogen signaling and collagen production [13]. The estrogen receptor α (ERα) is a nuclear receptor that is activated by the sex hormone estrogen. ERα is encoded by the gene estrogen receptor 1 (*ESR1*) which is widely studied to explore the gender difference in knee OA. *ESR1* is a ligand-activated transcription factor composed of several domains important for hormone binding [14]. The combination of *ESR1* and estrogen regulates the gene expression and gene function [15]. Therefore, the polymorphisms of *ESR1* have frequently been investigated for describing the genetic effect in OA.

*ESR1* includes eight exons and seven introns located at 6q25.1, and the most investigated polymorphisms are PvuII (rs2234693), BtgI (rs2228480), and Xbal (rs9340799). The polymorphisms BtgI [16] and PvuII [17] were confirmed as the risk factors of OA. However, the evidence of the Xbal polymorphism is still not sufficient for concluding its effect on knee OA [18]. Eight studies have investigated the association between Xbal polymorphism and knee OA until now [19,20,21,22,23,24,25,26]. However, no satisfactory consensus has been reached especially, in Asians. Three studies considered the major allele (A allele) carriers had higher risk in knee OA [19,23,24], but one study showed the significant risk effect of minor allele (G allele) [20]. Two studies also showed null association between *ESR1* Xbal polymorphism and knee OA [22,25]. The trial sequential analysis (TSA) provided an opportunity to evaluate whether the most recent conclusions are supported by the current cumulative samples [27]. The current sample size provided by current studies is only 2223 [19,20,22,23,24,25], which is not enough for obtaining a decisive conclusion. The aim of this study was to conduct a case–control study to validate the association *ESR1* Xbal polymorphism and knee OA in Taiwan. We also performed a meta-analysis to improve the evidence level and evaluate whether the latest conclusions are supported by the current cumulative samples using trial sequential analysis (TSA).

## 2. Materials and Methods

### 2.1. Case–Control Study

#### 2.1.1. Sample Size and Ethical Issues

This study was approved by the institutional review board (TSGH-1-104-05-006) of the Tri Service General Hospital (TSGH). Volunteers signed the consent form after the investigators had provided an explanation of the study.

#### 2.1.2. Subjects

We recruited the participants at the Health Management Centre of TSGH in a physical program from March 2015 to July 2018. The Taipei city senior medical check-up program is a governmental welfare program provided for people aged 65 years or older and who have been registered residents in Taipei city for more than one year. The exclusion criteria were as follows: (1) patients who had undergone knee surgery (e.g., total knee arthroplasty); and (2) those unable to provide a sufficient blood sample. Demographic data including age, gender, body mass index (BMI: kg/m^2^), systolic/diastolic blood pressure, and bone mineral density (BMD) were collected from medical records. BMD was calculated as T-score to define the osteoporosis. In total, 970 independent subjects aged 65 years or older participated in this study.

All participants underwent a radiographic examination of both knees with anterior–posterior and lateral views analyzed as well as weight bearing and foot-map positioning recorded. Knee radiographs were read and scored by our radiologist using the Kellgren–Lawrence (KL) grading system [28]. In the KL system, radiographs receive scores of 0–4 points. For patients with different KL grades in each knee, the more advanced grade was used for evaluation. We used a radiographic KL grade of ≥2 to define knee OA. According to the above classification, the study included 497 knee OA patients and 473 healthy controls.

#### 2.1.3. Genomic DNA Extraction and Genotyping

Medical technologists or nurses collected 5 mL of intravenous blood samples from each of the volunteers. Genomic DNA from peripheral blood samples was isolated using standard procedures for proteinase K (Invitrogen, Carlsbad, CA, USA) digestion and the phenol/chloroform method. *ESR1* Xbal was genotyped by iPLEX Gold SNP genotyping. Inter- and intra-replication validation was used to assess the genotyping experiment quality. Inter-replication validation was repeated for 100 samples (~10%), and the concordance rate was 100%.

#### 2.1.4. Statistical Analysis

Continuous variables of the general demographic data were expressed as mean and standard deviation using Student’s *t* test. Differences in genotype and allele frequencies between knee OA patients and healthy controls were tested using a χ^2^ test. Odd ratios (ORs) and 95% confidence intervals (CIs) for the risk of knee OA were calculated using logistic regression. Calculation of genetic polymorphism and knee OA risk was expressed using allele type, genotype, and dominant/recessive models. A *p* value of <0.05 was considered significant. R 3.4.4 was used for statistical analyses.

### 2.2. Meta-Analysis

#### 2.2.1. Search Methods and Criteria for Study Consideration

The PRISMA checklist and Meta-analysis on Genetic Association Studies Checklist are described in Table S1 [29]. Related terms of “*ESR1* Xbal” and “osteoarthritis” were used to search the PubMed and EMBASE for articles published up to 31 December 2018 (Table S2).

Moreover, the publications included in the meta-analysis studies were manually examined to avoid the omission of important articles. The inclusion criteria were as following: (1) cross-sectional surveys or case–control studies; (2) OA defined as a KL grade of ≥2; and (3) a detailed distribution of *ESR1* Xbal genotypes. If the published data were incomplete, we made attempts to contact the authors for further information.

#### 2.2.2. Data Extraction and Quality Assessment

Two reviewers (Yu-Hao Huang and Sui-Lung Su) independently extracted data and assessed the risk of bias. For each article, we recorded the first author’s name, year of publication, study design, ethnicity of the study population, definition of the case group, and population characteristics in cases and controls (proportion of male subjects, BMI, BMD, and *ESR1* Xbal genotype distribution). All extracted papers were assessed using the Newcastle–Ottawa Scale, and all received scores of >5 points.

#### 2.2.3. Statistical Analysis

The meta-analysis examined the association between *ESR1* Xbal polymorphisms and the risk of knee OA in each study using ORs with 95% CIs. The *I^2^* estimating by the DerSimonian–Laird method was used to assess heterogeneity, and more than 50% indicated a moderate to high heterogeneity [30]. All analyses were conducted by the random-effects model to obtain the summary results. Three common genetic models, including allele type, dominant, and recessive models, were used to calculate the association between genetic polymorphism and the risk of knee OA. The allele model was considered as the primary outcome. Egger’s regression and a funnel plot were used to test the symmetry of pooled results [31]. This study considered a *p* value of <0.05 to be significant for all analyses. Statistical analyses were conducted using the “meta” packages of R 3.4.4.

## 3. Results

### 3.1. Case–Control Study

In total, 970 elders participated in this study. The characteristics of knee OA cases and controls are shown in Table 1. In total, 497 cases with a mean age of 75.07 ± 7.54 years (168 men and 329 women) and 473 controls with a mean age of 73.66 ± 7.41 years (228 men and 245 women) were defined by the KL system. The mean age of cases was significantly higher than controls (*p* = 0.003), and being female was a significant risk factor on knee OA (*p* < 0.001). The cases had a higher mean BMI compared to the controls (24.78 ± 3.64 vs. 24.12 ± 3.28, *p* = 0.009), and it might be due to a lower mean height (157.77 ± 8.09 vs. 159.21 ± 7.90, *p* = 0.008) but not due to the weight (*p* = 0.977). The distributions of other characteristics were similar in cases and controls (*p* = 0.131 in systolic blood pressure, *p* = 0.546 in diastolic blood pressure, *p* = 0.257 in T-score, and *p* = 0.613 in osteoporosis). The results of gender-based stratification analysis are presented in Table S3.

We further used allele type, genotype, dominant, and recessive assumptions to test the association between *ESR1* XbaI and knee OA (Table 2). Nineteen samples could not be genotyped by our genotyping process, so there were only 951 (98.0%) samples included in the genetic analyses. The G allele frequencies were 20.8% and 22.1% in cases and controls, respectively. The G allele carries might not have higher risk of knee OA compared to the A allele carries in univariable analysis [Crude-OR: 0.97 (95% CI: 0.78–1.20)], and the result are similar after adjusting by gender, age, and BMI [Adj-OR: 0.90 (95% CI: 0.71–1.15)]. Moreover, we also evaluated the results from the genotype, dominant, and recessive models and only obtained nonsignificant results both before and after adjusting covariates. Table S4 shows the result of the data sent for a further stratification analysis, in which no association was found between *ESR1* XbaI and knee OA. Therefore, our case–control study showed a null association between *ESR1* XbaI and knee OA. We used meta-analysis to further enhance the level of evidence for exploring this issue.

### 3.2. Meta-Analysis

**Figure 1:**
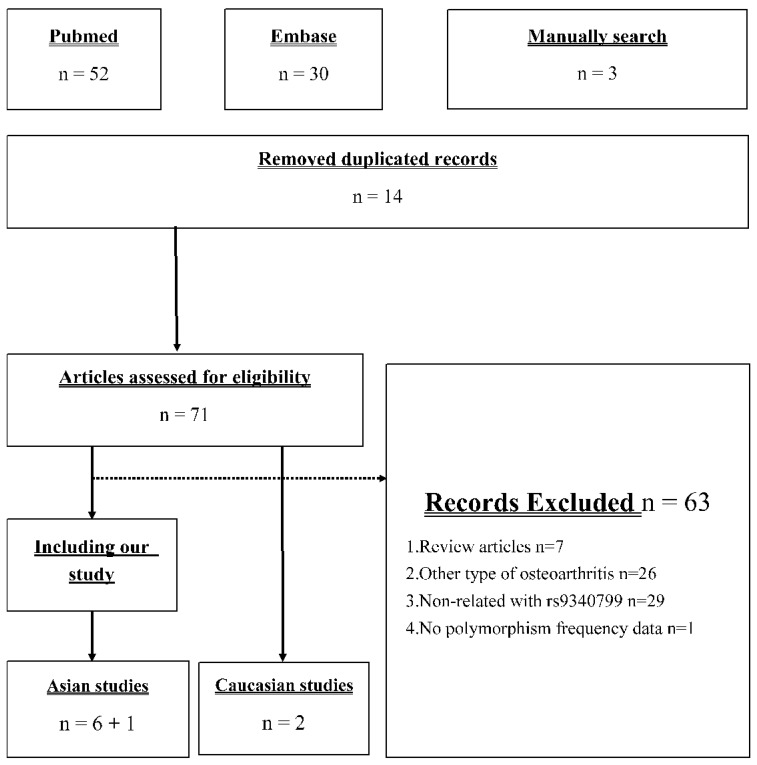
Flow diagram of the identification process for eligible studies.

Figure 1 presents the identification process in our meta-analysis. Our search strategy returned 52 and 30 records from PubMed and EMBASE, respectively. Moreover, there were three studies [22,23,24] included in the newest meta-analysis [18] but not searched. Seventy-one records remained after removing duplicate records, and only eight studies were used in further meta-analysis [19,20,21,22,23,24,25,26]. The 63 excluded articles included 7 review articles, 26 studies investigating other type of OA, 29 studies investigating the association between other polymorphism and knee OA, and 1 study provided insufficient information for analysis. Based on the current studies of this issue, we conducted a meta-analysis including our case–control study, six other Asian studies, and two Caucasian studies.

The detailed studies’ characteristics are presented in Table 3. The included studies were published during 2003–2014 before adding our samples. All included studies were case–control studies, and the definition of OA was based on KL scoring system. The Caucasian studies were conducted in the Netherlands and Mexico, respectively, and most Asian studies were from China. Table 4 shows the population information of included studies. Our study population had the lowest mean BMI. We used the detailed distribution of genotype in cases and control to conduct the following meta-analysis.

**Figure 2:**
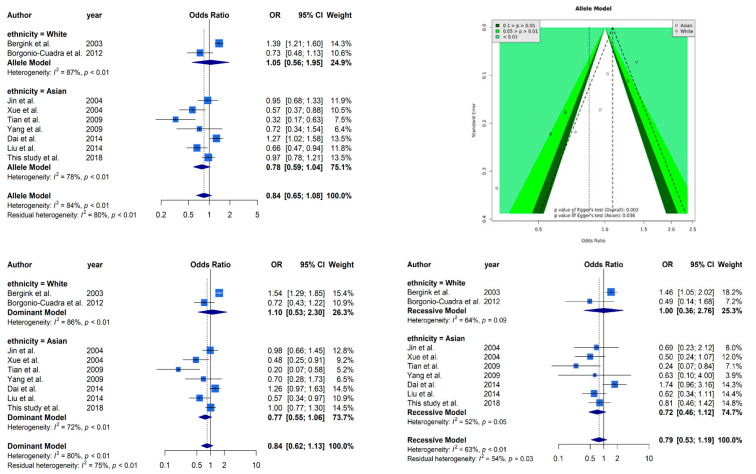
Selected results from the meta-analysis of *ESR1* Xbal and knee OA. The top left subplot is a forest plot based on an allele model assumption (reference: A allele) and the top right subplot is a funnel plot based on the allele model assumption. The results obtained with the dominant (AG + GG vs. AA) and recessive (GG vs. AA + AG) models are presented at the bottom. All results are nonsignificant.

Figure 2 presents selected results from our meta-analysis. The analysis based on allele model assumption showed a similar non-significant result [OR: 0.85 (95% CI: 0.66–1.09)] as our case–control study. The stratified analysis also showed the same trends in Caucasians [OR: 0.85 (95% CI: 0.66–1.09)] and Asians [OR: 0.85 (95% CI: 0.66–1.09)]. A funnel plot was used to demonstrate the association between ORs and standard error in the allele model, with each point representing a study. We found significant evidence of asymmetry from a visual observation, and Egger’s regression yielded the same result (*p* = 0.002 overall and *p* = 0.036 in the Asian subgroup). It is worth mentioning that only two Caucasian studies cannot be tested by Egger’s regression. High heterogeneity (*I^2^* = 83%) was observed in our meta-analysis, and the stratified analysis could not reduce the heterogeneity (*I^2^* = 87% in the Caucasian subgroup and *I^2^* = 78% in the Asian subgroup). The other analyses based on dominant and recessive model showed similar results as the allele model. Non-significant effects of *ESR1* Xbal and high heterogeneities were observed in all analyses.

### 3.3. TSA Evaluation

**Figure 3:**
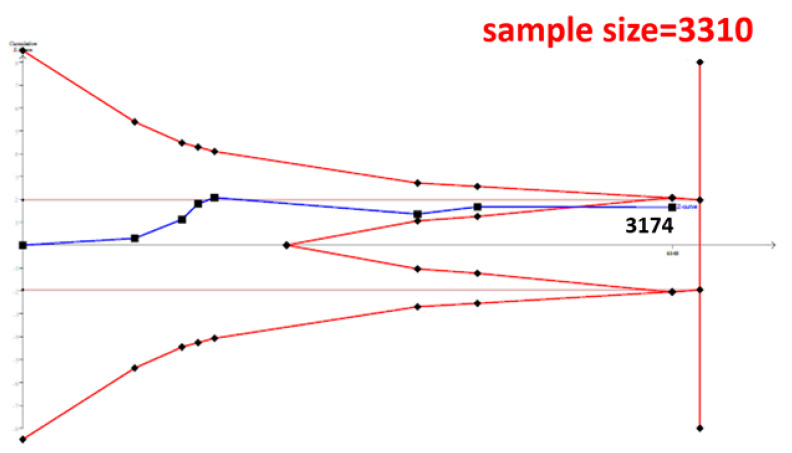
Trial Sequential Analysis (TSA) in Asians. We performed a TSA using an allele model assumption but replaced the allele count with the sample size (divided by 2). Detailed settings: Significance level = 0.05; Power = 0.8; ratio of controls to cases = 1; hypothetical proportion of controls with G allele = 0.19; least extreme OR to be detected = 1.5; *I^2^* (heterogeneity) = 80%.

**Figure 4:**
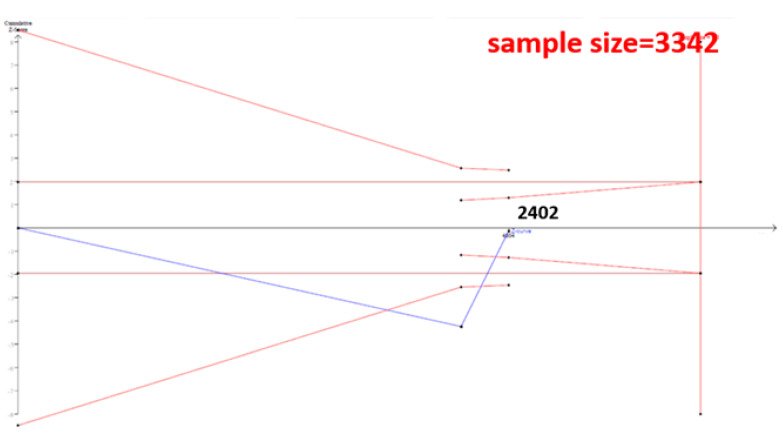
Trial Sequential Analysis (TSA) in Caucasians. We performed a TSA using an allele model assumption but replaced the allele count with the sample size (divided by 2). Detailed settings: Significance level = 0.05; Power = 0.8; ratio of controls to cases = 1; hypothetical proportion of controls with G allele = 0.31; least extreme OR to be detected = 1.5; *I^2^* (heterogeneity) = 80%.

The cumulative sample size for the Asian population was only 2223 before adding our case–control samples, which could not establish a decisive conclusion. Our case–control study provided 951 samples to enhance the information, and a total of 3174 allowed the Z curve to reach the futility area (Figure 3). This result shows that *ESR1* Xbal and knee OA are not significantly related in Asians, and a decisive conclusion could be confirmed. Our case–control study was the critical information in this meta-analysis. Figure 4 shows the result of TSA in Caucasians, and the cumulative sample size had exceeded the threshold value for concluding the same meaning. This meta-analysis showed the consistent non-significant results in all analyses.

## 4. Discussion

In summary, this meta-analysis showed the non-significant correlation between *ESR1* Xbal and knee OA both in Caucasians and Asians. TSA result show that the current cumulative samples were sufficient to reach a decisive conclusion and our case–control sample provided the critical information in Asians. The cumulative sample size of Caucasians was also sufficient to reach a decisive conclusion. To the best of our knowledge, this study is the first meta-analysis to explore the correlation between *ESR1* Xbal and knee OA using TSA.

Estrogen played a protective role in OA pathogenesis [32]. The mechanism of this protective effect was presented in cartilages. Previous studies showed the expression of ERα in cartilages [33] and osteoblasts [34], which inhibited the inflammatory cytokines [34]. Knee OA was also considered an inflammation-related disease [35], and previous studies confirmed a relationship between inflammation-related gene polymorphisms and knee OA [36,37]. As a matter of fact, most polymorphisms in *ESR1* (BtgI [16] and PvuII [17]) were confirmed as risk factors of OA. It seems that polymorphisms located in *ESR1* might be related to OA based on this pathway.

However, the *ESR1* Xbal polymorphism is located in intron, which is a nucleotide sequence within a gene removed by RNA splicing during maturation of the messenger RNA. Because the messenger RNA is the final production in transcription process for following protein translation, the expression of ERα might not be affected by the mutation in intron. Therefore, the intron polymorphism, *ESR1* Xbal, might also not be related with the above mechanism. TSA found that a decisive conclusion could be established and the samples in this study provided critical information. The non-significant results of our analyses might be reasonable, and we considered that candidate polymorphisms should be seriously picked out based on the molecular function.

Significant asymmetry was observed in our funnel plot, and the Egger’s regression yielded the same result. Some studies considered that this implies potential publication bias in the meta-analysis which might hazard the reliability of the conclusion. However, Egger refuted this opinion and pointed out four other reasons cause asymmetry: (1) true heterogeneity; (2) data irregularities; (3) choice of effect measure; and (4) chance [31]. Our search strategy was carefully designed to avoid publication bias. We manually examined the references of previous meta-analysis to obtain as complete publications as possible. Moreover, a high heterogeneity was observed in our meta-analysis, which was also presented in Asians and Caucasians. Therefore, the reason of significant asymmetry might be due to the true heterogeneity, and our conclusion of meta-analysis was reliable.

This study has three limitations. First, the high heterogeneity could not be explained, which might imply potential gene–gene and gene–environment interactions. We used the ethnicity as the stratified variable, but it could not reduce the heterogeneity. Our previous study developed a revised version of meta-regression, known as case-weighted meta-regression, to analyze the gene–gene and gene–environment interactions using average population information [38]. However, the information was not enough to use this method because only few studies provided the population information in their articles. We suggested further researchers should provide complete population characteristics for future meta-analysis. Second, we relied on tabular data rather than individual patient data, and the potential confounding factors could not be adjusted. However, this study provided a case–control sample for trying to adjust the confounding effects. The multivariable analysis was similar with the univariable analysis, representing minor or null confounding effects in the association between *ESR1* Xbal and knee OA. Third, only nine studies including our samples were used in our meta-analysis. The sample size of this polymorphism in *ESR1* was significantly less than other polymorphisms, although this was the largest study to date. TSA showed that the Z curve reached the futility area in both Asians and Caucasians, which represented that a decisive conclusion could be established. This explained that the needed sample size of the association between *ESR1* Xbal and knee OA might not need to reach the number in other polymorphisms.

In conclusion, the definitely null relationship between *ESR1* Xbal and knee OA was validated in this study. The information of Caucasian studies was sufficient before this study, but our case–control study provided the critical evidence to elaborate the same phenomenon in Asians. This study integrated all current evidence to conclude this null relationship between *ESR1* Xbal and knee OA for suggesting no necessity of future studies. Although the initial idea for investigating the *ESR1* Xbal on knee OA based on the *ESR1* signal pathway seems adequate, we considered that future candidate polymorphisms should be seriously chosen based on the complete molecular function. Functional analysis such as expression analysis of messenger RNA should be conduct before large-scale epidemiological studies. The only unexplained phenomenon in *ESR1* Xbal on knee OA was the high heterogeneities in both Caucasians and Asians. Future studies should further explore the source of heterogeneity, which might be hidden in unmeasured gene–gene and gene–environment interactions.

## Figures and Tables

**Table 1:** Characteristics of subjects with knee osteoarthritis and control subjects.

		Case (*N* = 497)	Control (*N* = 473)	*p* Value
**Gender**	Female	329 (66.2%)	245 (51.8%)	<0.001
	Male	168 (33.8%)	228 (48.2%)	
**Age (years)**		75.07 ± 7.54	73.66 ± 7.41	0.003
**Height (cm)**		157.77 ± 8.09	159.21 ± 7.90	0.008
**Weight (kg)**		61.21 ± 11.49	61.24 ± 10.64	0.977
**BMI (kg/m^2^)**		24.78 ± 3.64	24.12 ± 3.28	0.009
**SBP (mmHg)**		132.79 ± 17.65	130.92 ± 16.04	0.131
**DBP (mmHg)**		76.61 ± 10.20	77.08 ± 11.34	0.546
**T-score**		−0.51 ± 1.85	−0.37 ± 1.85	0.257
**Osteoporosis (%)**		54 (11.2%)	46 (10.2%)	0.613

BMI, body mass index; SBP, systolic blood pressure; DBP, diastolic blood pressure.

**Table 2:** The association between *ESR1* XbaI and knee OA in case–control study.

	Case	Control	Crude-OR (95% CI)	*p* Value	Adj-OR (95% CI) ^$^	*p*-Value
**Allele**						
A allele	769 (79.1%)	731 (77.7%)	1		1	
G allele	203 (20.8%)	199 (22.1%)	0.97 (0.78–1.20)	0.790	0.90 (0.71–1.15)	0.779
**Genotype**						
AA	307 (63.1%)	294 (63.2%)	1		1	
AG	155 (31.8%)	143 (30.7%)	1.04 (0.79–1.37)	0.792	0.97 (0.72–1.32)	0.857
GG	24 (4.9%)	28 (6.0%)	0.82 (0.47–1.45)	0.496	0.79 (0.42–1.47)	0.448
**Dominant**						
AA	307 (63.1%)	294 (63.2%)	1		1	
AG + GG	179 (36.8%)	171 (36.7%)	1.00 (0.77–1.30)	0.985	0.94 (0.71–1.26)	0.687
**Recessive**						
AG + AA	462 (94.9%)	437 (93.9%)	1		1	
GG	24 (4.9%)	28 (6.0%)	0.81 (0.46–1.42)	0.463	0.79 (0.43–1.47)	0.460

OR, odds ratio; ^$^ adjusted by gender, age, and body mass index.

**Table 3:** Studies’ characteristics in the meta-analysis.

First Author	Year	Country	Ethnicity	Study Design	Definition of OA
Bergink [26]	2003	Netherland	Caucasian	case–control study	KL ≥ 2
Jin [25]	2004	Korea	Asian	case–control study	KL ≥ 2
Xue [24]	2004	China	Asian	case–control study	KL ≥ 2
Tian [23]	2009	China	Asian	case–control study	KL ≥ 2
Yang [22]	2009	China	Asian	case–control study	KL ≥ 2
Borgonio-Cuadra [21]	2012	Mexico	Caucasian	case–control study	KL ≥ 2
Dai [20]	2014	China	Asian	case–control study	KL ≥ 2
Liu [19]	2014	China	Asian	case–control study	KL ≥ 2
This study	2018	Taiwan	Asian	case–control study	KL ≥ 2

**Table 4:** The population information of included studies.

First Author	Year	Male (%) ^#^	Mean BMI (kg/m^2^) ^#^	Mean T-Score ^#^	Genotype Frequencies in Cases ^$^	Genotype Frequencies in Controls ^$^
Bergink [26]	2003	28%/45%	27.3/25.7	0.86/0.82	643/682/158	372/263/52
Jin [25]	2004	35%/46%	25.2/NA		98/49/4	256/126/15
Xue [24]	2004				21/24/10	40/82/54
Tian [23]	2009				18/16/4	6/21/13
Yang [22]	2009				28/11/2	24/13/3
Borgonio-Cuadra [21]	2012	20%/18%	26.5/25.6		70/41/4	62/47/8
Dai [20]	2014	25%/77%	26.1/24.3		288/152/29	348/155/19
Liu [19]	2014		31.2/24.1	0.75/0.66	36/43/19	49/92/55
This study	2018	48%/34%	24.1/24.7	−0.37/−0.51	307/155/24	294/143/28

^#^ The data are shown in the order of control/case; ^$^ the genotype frequencies are shown in the order of AA/AG/GG.

## Supplementary Materials

The following are available online at https://www.mdpi.com/2073-4425/12/3/404/s1, Table S1: PRISMA 2009 Checklist, Table S2: Search strategies of meta-analysis, Table S3: Characteristics of gender stratified subjects with knee osteoarthritis and control subjects, Table S4: The association between *ESR1* XbaI and knee OA in gender-stratified case–control study.
