# Supplementary material for: The Decisive Case-Control Study Elaborates the Null Association between ESR1 XbaI and Osteoarthritis in Asians: A Case–Control Study and Meta-Analysis
Source: Genes (Basel). 2021 Mar 12;12(3):404. doi: 10.3390/genes12030404 (PMC7999595; doi:10.3390/genes12030404)
Supplement: Supplementary file 1 [file genes-12-00404-s001.zip › genes-1094497 supplementary/supplementary Table S2.docx]

搜尋關鍵字及網址

| **Relevant text of ESR1 XbaI**   1. polymorphism, genetic 2. polymorphism 3. genetic 4. genetic polymorphism 5. SNP 6. Single Nucleotide Polymorphism 7. polymorphism 8. ESR1 9. Estrogen Nuclear Receptor 10. Estrogen Receptor Type I 11. Estrogen Receptors 12. Estrogen Receptors Type I 13. Receptor, Estrogen Nuclear 14. Receptors, Estrogen, Type I | **Relevant text of osteoarthritis**   1. OA 2. osteoarthritis 3. Arthritis, Degenerative 4. Osteoarthrosis 5. Osteoarthrosis Deformans 6. knee osteoarthritis |
| --- | --- |

Web sites and uniform resource locator :

**MeSHBrowser**: <http://www.nlm.nih.gov/mesh/MBrowser.html>

**PubMed**: <http://www.ncbi.nlm.nih.gov/pubmed>

**Embase**: https://www.embase.com
