# Supplementary material for: The Decisive Case-Control Study Elaborates the Null Association between ESR1 XbaI and Osteoarthritis in Asians: A Case–Control Study and Meta-Analysis
Source: Genes (Basel). 2021 Mar 12;12(3):404. doi: 10.3390/genes12030404 (PMC7999595; doi:10.3390/genes12030404)
Supplement: Supplementary file 1 [file genes-12-00404-s001.zip › genes-1094497 supplementary/supplementary Table S3.docx]

**S3 Table. Characteristics of gender stratified subjects with knee osteoarthritis and control subjects**

|  |  | **Case (N = 497)** | **Control (N = 473)** | **p value** |
| --- | --- | --- | --- | --- |
| **Gender** | Female | 329(66.2%) | 245(51.8%) |  |
| **Age (years)** |  | 74.22±6.71 | 71.60±5.88 | <0.001 |
| **Height (cm)** |  | 153.82±5.95 | 154.04±5.40 | 0.664 |
| **Weight (kg)** |  | 56.92±9.31 | 56.05±8.43 | 0.271 |
| **BMI (kg/m^2^)** |  | 24.07±3.79 | 23.65±3.59 | 0.200 |
| **SBP (mmHg)** |  | 133.05±18.37 | 130.25±16.80 | 0.101 |
| **DBP (mmHg)** |  | 76.67±10.08 | 76.02±10.98 | 0.516 |
| **T-score** |  | -1.20±1.30 | -1.33±1.36 | 0.263 |
|  |  |  |  |  |
| **Gender** | Male | 168(33.8%) | 228(48.2%) |  |
| **Age (years)** |  | 76.72±8.72 | 75.86±8.22 | 0.316 |
| **Height (cm)** |  | 165.55±5.80 | 164.97±6.06 | 0.370 |
| **Weight (kg)** |  | 69.71±10.65 | 67.05±9.83 | 0.016 |
| **BMI (kg/m^2^)** |  | 25.41±3.42 | 24.58±2.99 | 0.018 |
| **SBP (mmHg)** |  | 132.25±16.12 | 131.62±15.23 | 0.731 |
| **DBP (mmHg)** |  | 76.47±10.47 | 78.18±11.62 | 0.189 |
| **T-score** |  | 0.87±1.99 | 0.68±1.74 | 0.331 |

BMI: body mass index; SBP: systolic blood pressure; DBP: diastolic blood pressure.
