# Supplementary material for: The Decisive Case-Control Study Elaborates the Null Association between ESR1 XbaI and Osteoarthritis in Asians: A Case–Control Study and Meta-Analysis
Source: Genes (Basel). 2021 Mar 12;12(3):404. doi: 10.3390/genes12030404 (PMC7999595; doi:10.3390/genes12030404)
Supplement: Supplementary file 1 [file genes-12-00404-s001.zip › genes-1094497 supplementary/supplementary Table S4.docx]

**S4 Table.** **The association between ESR1 XbaI and knee OA in gender-stratified case-control study.**

|  | **Case** | **Control** | **Crude-OR (95%CI)** | **p value** | **Adj-OR (95%CI)^$^** | **p-value** |
| --- | --- | --- | --- | --- | --- | --- |
| **Female**  **Allele** |  |  |  |  |  |  |
| A allele | 374(77.3%) | 520(80.5%) | 1 |  | 1 |  |
| G allele | 110(22.7%) | 126(19.5%) | 0.82 (0.62 - 1.10) | 0.188 | 0.79 (0.58 - 1.08) | 0.136 |
| **Genotype** |  |  |  |  |  |  |
| AA | 149(61.6%) | 210(65.0%) | 1 |  | 1 |  |
| AG | 76(31.4%) | 100(31.0%) | 0.93 (0.65 - 1.34) | 0.712 | 0.88 (0.60 - 1.30) | 0.524 |
| GG | 17(7.0%) | 13(4.0%) | 0.54 (0.26 - 1.15) | 0.111 | 0.52 (0.23 - 1.17) | 0.113 |
| **Dominant** |  |  |  |  |  |  |
| AA | 149(61.6%) | 210(65.0%) | 1 |  | 1 |  |
| AG+GG | 93(38.4%) | 113(35.0%) | 0.86 (0.61 - 1.22) | 0.400 | 0.82 (0.56 - 1.18) | 0.282 |
| **Recessive** |  |  |  |  |  |  |
| AG+AA | 225(93.0%) | 310(96.0%) | 1 |  | 1 |  |
| GG | 17(7.0%) | 13(4.0%) | 0.56 (0.26 - 1.17) | 0.120 | 0.54 (0.24 - 1.21) | 0.133 |
|  |  |  |  |  |  |  |
| **Male**  **Allele** |  |  |  |  |  |  |
| A allele | 357(80.0%) | 249(76.4%) | 1 |  | 1 |  |
| G allele | 89(20.0%) | 77(23.6%) | 1.24 (0.88 - 1.75) | 0.221 | 1.20 (0.83 - 1.75) | 0.337 |
| **Genotype** |  |  |  |  |  |  |
| AA | 145(65.0%) | 97(59.5%) | 1 |  | 1 |  |
| AG | 67(30.0%) | 55(33.7%) | 1.23 (0.79 - 1.90) | 0.362 | 1.20 (0.74 - 1.93) | 0.463 |
| GG | 11(4.9%) | 11(6.7%) | 1.49 (0.62 - 3.58) | 0.368 | 1.40 (0.54 - 3.63) | 0.491 |
| **Dominant** |  |  |  |  |  |  |
| AA | 145(65.0%) | 97(59.5%) | 1 |  | 1 |  |
| AG+GG | 78(35.0%) | 66(40.5%) | 1.26 (0.83 - 1.92) | 0.269 | 1.23 (0.78 - 1.93) | 0.379 |
| **Recessive** |  |  |  |  |  |  |
| AG+AA | 212(95.1%) | 152(93.3%) | 1 |  | 1 |  |
| GG | 11(4.9%) | 11(6.7%) | 1.39 (0.59 - 3.30) | 0.449 | 1.32 (0.51 - 3.38) | 0.564 |

OR: odds ratio; ^$^: adjusted by age, and body mass index.
